# The Co-development of Friends’ Delinquency with Adolescents’ Delinquency and Short-term Mindsets: The Moderating Role of Co-Offending

**DOI:** 10.1007/s10964-021-01417-z

**Published:** 2021-04-21

**Authors:** Ivy N. Defoe, Jean-Louis van Gelder, Denis Ribeaud, Manuel Eisner

**Affiliations:** 1 Forensic Child and Youth Care Sciences, University of Amsterdam, 1001 NG Amsterdam, The Netherlands; 2 Max Planck Institute for Foreign and International Criminal Law, Freiburg, Germany; 3 University of Zurich, Zurich, Switzerland; 4 University of Cambridge, Cambridge, UK

**Keywords:** Peer delinquency, Co-offending, Impulsivity, School future orientation, Delinquency, Adolescence

## Abstract

*The companions in crime hypothesis* suggests that co-offending moderates the link between peer delinquency and adolescent delinquency. However, this hypothesis has rarely been investigated longitudinally. Hence, this study investigated the co-development of friends’ delinquency and adolescents’ delinquency, as well as the co-development of friends’ delinquency and short-term mindsets (impulsivity and lack of school future orientation). Whether this co-development is stronger when adolescents engage in co-offending was also investigated. Three data waves with two year lags from an ethnically-diverse adolescent sample (at wave 1: *N* = 1365; 48.6% female; *M*_age_ = 13.67; age range = 12.33–15.09 years) in Switzerland were used. The results from parallel process latent growth modeling showed that the co-development between friends’ delinquency and adolescents’ delinquency was stronger when adolescents engaged in co-offending. Thus co-offending likely provides direct access to a setting in which adolescents continue to model the delinquency they learned with their peers.

## Introduction

Peer affiliation and, in return, “peer influence” are regarded as hallmarks of adolescence (Brechwald and Prinstein 2011). Indeed, the finding that delinquency of peers predicts adolescent delinquency has been consistently replicated (Brechwald and Prinstein 2011). However, it has rarely been investigated whether delinquency of peers is also associated with other correlates of delinquency such as short-term mindsets (e.g., impulsivity and lack of future orientation). Furthermore, it is often presumed that delinquent peer affiliation implies that adolescents are also committing delinquency together *with* their peers (i.e., co-offending) (Brechwald and Prinstein 2011; Warr 2002). In fact, some theorists posit that engaging in delinquency with peers is a primary mechanism that causes the progression of delinquency during adolescence (Warr 2002). It can be extrapolated from this hypothesis that co-offending moderates the link between peer delinquency and adolescent delinquency. Yet, co-offending is rarely measured directly in the (developmental) psychology literature, and longitudinal studies that directly measure this concept in the criminological literature are also uncommon (cf Goldweber et al. 2011). As a result, longitudinal studies are virtually non-existent on whether co-offending indeed plays a moderating role in the relationship between delinquency on the one hand and delinquency of peers and short-term mindsets on the other hand. To this end, drawing upon the companions in crime hypothesis (Warr 2002), differential association theory (Sutherland 1947) and the psychosocial maturity hypothesis (Steinberg et al. 2009), the current longitudinal study investigates whether the co-development of (non-)best friends’ delinquency with adolescents’ delinquency and short-term mindsets (impulsivity and lack of school future orientation) are stronger when adolescents engage in co-offending with their friends.

### Co-development of Friends’ Delinquency and Adolescent Delinquency

The peer context becomes increasingly important for adolescents. In addition to close (smaller in size) friendships with best friends, individuals interactions with peers also occur in cliques, crowds, and larger networks of friends (Brechwald and Prinstein 2011). Research is currently mixed on the influences of best-friends versus such ancillary friends (i.e., non-best friends; Rees and Pogarsky 2011). Adolescents tend to overestimate the similarity between their own behavior and the behavior of their friends’ behavior when perceptual measures of peer delinquency are used (Rees and Greg Pogarsky 2011). Nevertheless, when accounting for measurement issues (e.g., the use of perceptual measures), “peer influence” on delinquent behavior has been shown to be paramount, as it can occur in comparable ways within adolescents’ interactions with delinquent best friends versus delinquent ancillary friends (Rees and Greg Pogarsky 2011).

When (delinquent) peers interact, co-offending can be the result, especially during adolescence (for an overview see: Warr 2002). Research on such delinquent peer influence suggests that it need not exclusively occur within (best) friend relationships, as it is common in broader peer networks as well (Brechwald and Prinstein 2011; Rees and Pogarsky 2011). Moreover, co-offending with delinquent peers has been theorized to link delinquent peer affiliation to adolescent delinquency (Dynes et al. 2015). Scholars have even proposed that “the age distribution of crime, stems from age related changes in peer relations” (p. 99; Warr 2002; but see Stolzenberg and D’Alessio 2008). This assumption has more recently been referred to as the “companions in crime hypothesis” (see Stolzenberg and D’Alessio 2008). This hypothesis suggests that peer influence during co-offending is a mechanism that causes the progression of delinquency during adolescence. For example, adolescents tend to imitate each other’s delinquency (Piquero and Moffitt 2010), and this effect can be even stronger when they are in company of each other while engaging in delinquency.

Although developmental theories and longitudinal research on co-offending is limited (cf. Goldweber et al. 2011), some criminological theories on social learning explicitly hypothesize that co-offending is associated with the onset, persistence and desistance of delinquency (Piquero et al. 2007). For instance, it is presumed that co-offending provides a setting wherein peers can directly influence each other and promote increasing levels of delinquency (Dynes et al. 2015). Individuals who co-offend might also be more susceptible to delinquent peer norms (Dynes et al. 2015). This assertion is in line with one of the propositions of differential association theory, which hypothesizes that that interactions with delinquent peers facilitate the learning of criminal techniques (Dynes et al. 2015; Sutherland 1947). Hence, it is assumed that this transmission of criminal techniques will be stronger if individuals are in the presence of each other during the engagement in delinquency (i.e., co-offending), versus whether individuals merely affiliate with delinquent peers (e.g., Dynes et al. 2015). Such co-offending could also be a result of peer group conformity (Asch 1951), and it may even influence (subsequent) delinquent behavior in the long-term. Taken together, it stands to reason that the influence of peer delinquency could be stronger for adolescents who co-offend compared to solo-offenders who merely have delinquent peers but do not co-offend with them. However, studies that could address this question are presently lacking (cf., Dynes et al. 2015), particularly because studies that assess delinquent peer affiliation do not specifically assess co-offending (cf. Dynes et al. 2015; McGloin and Stickle 2011).

Nevertheless, at least two cross-sectional studies based on court-involved youth have investigated related questions. One of those studies found that the link between friends’ delinquency and adolescent delinquency only existed for adolescents who actually co-offend with their peers (Dynes et al. 2015). Additionally, the second study reported that compared to other offenders, chronic offenders were less likely to mention “peer influence” as a reason for engaging in delinquency (McGloin and Stickle 2011). However, these two offender groups were equally likely to engage in co-offending with peers (McGloin and Stickle 2011). It was thus concluded that although chronic offenders are less likely to engage in delinquency because of their peers, they are still just as likely to engage in co-offending (McGloin and Stickle 2011). These results suggest that delinquency of peers does not necessarily have to imply that adolescents are engaging in delinquency with their peers, and that delinquency with peers can moderate the link between peer delinquency and adolescent delinquency. Building on these two cross-sectional studies on court-involved youth, an important aim of the current study is to use a longitudinal design to establish whether there is co-development among adolescents’ delinquency and best friends’ delinquency in a community sample. Furthermore, the current study uniquely examines whether this hypothesized co-development is stronger when adolescents co-offend with their friends. These hypotheses are in line with the companions in crime hypothesis (Warr 2002).

### Co-development of Friends’ Delinquency and Short-term Mindsets

The psychosocial maturity hypothesis suggests that influence of peers on (deviant) behaviors, and indicators of short-term mindsets (e.g., impulsivity, lack future orientation), all show significant and similar non-linear development particularly during adolescence, with these behaviors peaking during mid-adolescence (Steinberg, 2008; Steinberg et al. 2009; but see e.g., Chen 2009; Duell et al. 2016). Extrapolating from this notion, the current study pioneers a test of whether the mere delinquency of best friends is developmentally interrelated with impulsivity and lack of school future orientation during adolescence, and whether this is particularly the case when adolescents engage in delinquency together. This hypothesis also aligns with differential association theory on transmission of attitudes and beliefs (Sutherland 1947; but see Hochstetler et al. 2002). Namely, exposure to values and beliefs about violation of the law via delinquent peers could promote short-term mindsets such as impulsivity and lack of future orientation. This hypothesis is important to investigate, because if peer delinquency and short-term mindsets show such co-development, this could imply that peer delinquency is related to adolescent delinquency *because* it contributes to the development of short-term mindsets in youth. As such, short-term mindsets could be the conduit through which the association between peer delinquency and adolescent delinquency develops over time. Essentially, adolescents may adapt their own attitudes from observing the impulsive and risky behavior of their delinquent peers, for example by hearing them talk about taking risks and disregarding the future (Meldrum et al. 2012).

Co-offending (i.e., being present and thus observing others engaging in delinquency) could likely also make the association between delinquency of peers and short-term mindsets stronger. Specifically, co-offending could lead adolescents to conclude that they must be impulsive individuals who do not care about the future—and this could thus encourage the development of short-term mindsets. As such, it is further conceivable that co-offending would exacerbate the link between delinquency of peers and adolescent delinquency, which the current study explicitly investigates. These assertions overlap with the three hypotheses mentioned earlier, namely, companions in crime hypothesis, psychosocial maturity hypothesis, and differential association theory. Then again, of note is that other scholars (Hochstetler et al. 2002) have been more critical of such assertions related to differential association which posits that crime-condoning tendencies are transmitted during co-offending. It has for example been argued that although interactions with delinquent peers during co-offending have shown to exacerbate delinquency, this is not because of increases in crime-condoning attitudes (Hochstetler et al. 2002). Namely, empirical research demonstrated that crime-condoning attitudes do not appear to be the mechanism that links delinquent peer influences into solo-offending or co-offending, which questions whether group influences such as co-offending is the mechanism of differential association (Hochstetler et al. 2002). However, a search of the current literature did not result in any studies that have explicitly investigated this implied interaction between delinquency of peers and co-offending in the prediction of crime-condoning tendencies such as impulsivity and lack of future orientation. Nevertheless, three longitudinal studies were located that investigated whether an indicator of peer delinquency is longitudinally associated with levels of impulsivity, or self-control, more broadly.

The first study (Meldrum et al. 2012) used a social network-design and showed that changes in delinquency of classmates were related to subsequent changes in adolescent self-control (see also: Huijsmans et al. 2019; Mcgloin and Shermer 2009). Hence, this study showed support for differential association theory (Akers 2008; Meldrum et al. 2012; Sutherland 1947). A fixed-effects model in combination with a sequential latent growth model (LGM) was used for the analyses (Meldrum et al. 2012), which could account for unmeasured time-stable correlates and identify a specific temporal ordering, respectively. The results showed that delinquent classmates encourage lower self-control in adolescents (Meldrum et al. 2012). However, since a sequential LGM was used, it is unclear whether *reverse* temporal-ordering between constructs might also exist. That is, individuals with lower levels of self-control might also be the ones who gravitate more towards delinquent peers. In order to facilitate the testing of such bi-directional links, a recent study based on the current study sample used cross-lagged panel modeling and showed that a broad measure of self-control (including impulsivity, self-centeredness, risk seeking, short temperedness, and preference for physical activities) was bi-directionally related to friends’ delinquency during different stages of adolescence (Huijsmans et al. 2019). However, none of the two abovementioned studies (i.e., Huijsmans et al. 2019; Meldrum et al. 2012) investigated whether peer delinquency and self-control “travel together” and show co-development over time. To address this possibility, the current study uses a *parallel* LGM approach. Additionally, unlike the current study, both of the aforementioned studies (Huijsmans et al. 2019; Meldrum et al. 2012) only assessed delinquency of peers, and not whether adolescents were actually engaging in co-offending. The current study adds to literature by additionally investigating co-offending, and whether it moderates the hypothesized co-development between delinquency of best friends and short-term mindsets—as extrapolated from a combination of the abovementioned three theories that guide this study.

The final relevant study did assess co-offending, although it did not explore it as a moderator (Goldweber et al. 2011; see also Ashton et al. 2020). Via trajectory group modeling among a sample of serious male adolescent offenders, it was demonstrated that the group which increasingly engaged in co-offending showed less psychosocial maturity (i.e., *more* short-term mindsets) versus the occasional/mixed solo-offenders and the exclusively solo offenders from age 14 to 17. Those results show support for psychosocial maturity hypothesis and differential association theory. The current study extends the aforementioned study (Goldweber et al. 2011) in various ways. First, in addition to including a measurement of co-offending, the present study simultaneously investigates whether mere delinquency of best friends also predicts short-term mindsets and whether this is dependent on whether adolescents are actually engaging in co-offending with their friends. That is, whether co-offending (versus non co-offending) serves as a moderator in the co-development between delinquency of best friends and short-term mindsets is investigated. Secondly, instead of using one measurement wave of short-term mindsets and group trajectory modeling, the present study uses three measurement waves of both short-term-mindsets and friends’ delinquency. This is achieved via parallel LGMs in order to investigate whether the development of these constructs show correlated change (i.e., co-development) over three waves. Thirdly, the current research questions are examined using a community sample as opposed to a clinical sample (see Goldweber et al. 2011).

Finally, unlike the similar abovementioned study (Goldweber et al. 2011), the current study takes well-established correlated risk factors of delinquency into account. For example, it has consistently been demonstrated that demographic factors such as gender and ethnicity are strong correlates of crime, namely males and ethnic minorities are over-represented in the juvenile justice system (for a review see: Piquero et al. 2015). Additionally, an extensive review of longitudinal studies found that socioeconomic factors, such as low family income (which is intertwined with parental occupation) as well as youth’s own school achievement are among the strongest predictors of juvenile delinquency (Murray and Farrington 2010). These important correlates will be controlled for in the current study.

## Current Study

Extrapolating from the companions in crime hypothesis, social learning theories (e.g., differential association theory) and the psychosocial maturity hypothesis and empirical studies (e.g., Dynes et al. 2015), it is to be expected that peer delinquency on the one hand shows co-development with adolescent delinquency and short-term mindsets on the other. This co-development is expected to be stronger when adolescents engage in co-offending. However, to date, there is a dearth of longitudinal studies that have investigated these hypotheses, and longitudinal studies on whether co-offending indeed plays a moderating role in the above-mentioned co-development links are virtually non-existent. The current study tests these research questions using three waves of longitudinal data from an ethnically-diverse Swiss community sample of adolescents, while controlling for ethnicity, gender, educational track, and socioeconomic status. The main hypothesis is that co-development of best friends’ delinquency with adolescent delinquency and short-term mindsets will exist and that these links will be stronger for youth who engage in co-offending.

## Methods

### Participants

The adolescents in the current study are participants in an 8-wave ongoing longitudinal-intervention study: “*Zurich Project on the Social Development from Childhood into Adulthood* (z-proso)”, which began in 2004, in Switzerland (Eisner et al. 2011). The data-collections took place every two years. In total, 1675 first graders (*M*_age_ = 7.45, SD = 0.39; 48% female) from 56 randomly selected schools formed the target sample at baseline (W1) (Van Gelder et al. 2015). Of these participants, 46% of both parents were not born in Switzerland (Van Gelder et al. 2015). Further detailed demographic information is reported in “Eisner et al. (2011)”. The current study is based on waves W5 to W7, because these waves overlap with the adolescence period, and because data on the main variables of interest were consistently collected across these waves. Accordingly, henceforth, W5 will be referred to as baseline (i.e., T1; *N* = 1365; 48.6% female). For W5 and W6, passive parental consent along with active informed participant consent (W5–W6) was obtained, whereas for W7 only active informed participant consent was obtained. From T1–T3, 82, 86, and 78% of the adolescents from the original target sample participated, respectively. The average ages were 13.67, 15.44, and 17.45 years from T1–T3, respectively. Paper questionnaires were filled out during leisure time in classroom-settings. Participants received an incentive worth the equivalent of US$30, US$50, and US$60 from T1–T3, respectively.

### Measures

#### Impulsivity

Participants reported on two items on the “impulsivity subscale” of an adjusted and abbreviated Self-control Scale of Grasmick et al. (1993) (Ribeaud and Eisner 2006). The items were: (1) I often act on the spur of the moment without stopping to think and (2) I often do whatever brings me pleasure here and now, even at the cost of some distant goal. Answer categories ranged from “fully untrue” (=1) to “fully true” (=4). This sub-scale has been validated in previous studies (Van Gelder et al. 2018, 2020). Mean scores were computed from the items on the scale. The Cronbach’s Alpha is biased towards scales with few items, hence the mean inter-item correlations is opted for to evaluate reliability. These mean inter-item correlations were 0.273, 0.275, 0.332 from T1–T3, respectively, which denotes adequate reliability (see Clark and Watson 1995).

#### School Future Orientation

Participants reported on the following three statements concerning school future orientation in the /school domain: (1) When I grow up I want to have an interesting job, and I’m doing everything now to work towards that goal, (2) I try hard at to have a good job later in life and (3) Doing well at is important to me. Answer categories ranged from “fully untrue” (=1) to “fully true” (=4) (Van Gelder et al. 2018). Mean scores were computed. The mean inter-item correlations were adequate (*r* = 0.472, 0.524, and 0.494, from T1–T3 respectively).

#### Delinquency

Participants reported on their engagement in 14 different delinquent behaviors within the past-12-months via a delinquency questionnaire adjusted from Wetzels et al. (2001). Items included both minor delinquency (graffitiing, vandalism) and more serious forms of delinquency (e.g., robbery, assault). Mean scores were computed from the yes/no responses on these items. Cronbach alphas were 0.774, 0.757, and 0.710 across T1–T3, respectively, indicating adequate reliability.

#### Delinquency of Friends

Participants were asked to answer the following questions about each of their two best friends: (1) in the last year, has he/she purposely hit or kicked another adolescent and injured them in the process? and (2) in the last year, has he/she stolen something from a shop, kiosk, or shopping mall? From the yes/no responses, the mean scale score of each best friend, was combined into one overall mean score that represented an average delinquency score of the adolescent’s two best friends. The mean inter-item correlations were 0.329, 0.257, 0.265 across T1–T3, denoting adequate reliability. However, of note is that the current reports of friends’ delinquency are based on a “perceptual measure” of best-friends’ delinquency. That is, the best friend is not reporting on his/her own delinquency. Nevertheless, since best friends form close companionships (versus individuals in the broader peer network), it is assumed that adolescent’s evaluations of their best-friends behavior are reliable (Rees and Pogarsky 2011).

#### Co-offending with Friends

Participants reported whether they ever engage in the following together with their friends: (1) meet up with friends and have fights with other adolescents, and (2) meet up with friends and together steal something from a shop or kiosk. Answer categories ranged from: “never” (=1) to “(almost) every day” (=6). Mean scores were computed, and the inter-item correlations showed adequate reliability: 435, 0.338, 0.402 from T1–T3 respectively.

Additionally a categorical variable that depicted 0 = “non co-offending group” (i.e., individuals who do not have experience with co-offending across all the three waves of the study) and 1 = “co-offending group” (i.e., individuals who have experience with co-offending at least 1 time across the 3 waves) was computed. This categorical variable was used in the multi-group analyses to investigate whether experience with co-offending serves as a moderator in the current study (see the strategy of analyses).

#### Control Variables

Gender (“0” for females and “1” for males), ethnicity (“0” = at least one parent born in Switzerland; “1” = two foreign-born parents), socio-economic status (SES) and educational track were included the parallel process LGM’s as control variables. The highest International Occupational Status (ISEI) score (Ganzeboom et al. 1992) of the two caregivers was used as an indicator of SES. Educational track at T1 included the following categories: 0 = special needs *or* primary school (e.g., participants who repeated (a) grade(s) in primary school); 1 = tracks leading to “blue-collar” apprenticeship (“Sekundarschule B/C”); 2 = tracks leading to “white-collar” apprenticeship (“Sekundarschule A”); 3 = tracks leading to university (“Mittelschule/Gymnasium”, i.e., A-levels equivalents).

### Strategy of Analyses

To model interrelations between individuals’ developmental trajectories for the constructs of interest over time, parallel process LGM’s in Mplus 8 were used. (Muthén and Muthén, 1998–2017). Using this methodology, an “intercept” (an individual’s *initial or baseline level* on a construct), and the slope (an individual’s rate and direction of change, i.e., their growth/development for a construct) are identified. Together these “growth factors” determine the level and shape of the developmental trajectories (Bollen and Curran 2006).

**Fig. 1:**
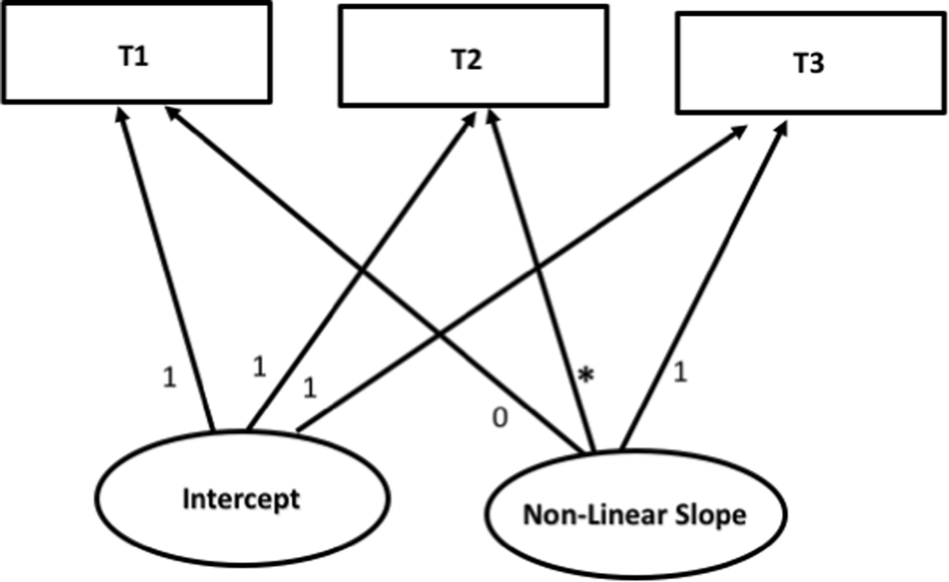
Conceptual diagram of an unspecified non-linear LGM

Prior to running the parallel process LGM’s, as a preliminary step a linear and non-linear unspecified growth curves (Meredith & Tisak, 1990) were run using a univariate LGM for each variable of interest (cf. Bollen and Curran 2006). So-called “unspecified” non-linear LGM’s are similar to specified non-linear LGM’s (e.g., a quadratic LGM or a cubic LGM), but with at least one of the slope loadings freely estimated (see Fig. 1) (S. C. Duncan et al. 1998; T. E. Duncan and Duncan 2009). As outlined in Bollen and Curran (2006); (see also Little 2013), the middle (T2) loading was freely estimated, while constraining the first (T1) slope loading to 0, and the last (T3) loading to 1.

In a second preliminary step, the AIC and Sample-size Adjusted Bayesian Information Criterion (SABIC; Akaike 1987; Sclove 1987) were used to compare the fit between the abovementioned linear and the unspecified non-linear univariate LGM’s. Subsequently, the best fitting model was retained for the main parallel process LGM analyses (Bollen and Curran 2006). Finally, in the parallel process LGM’s, baseline levels of gender, SES, educational track, and ethnicity were controlled for by including these variables in the model as predictors of the growth factors.

In sum, a total of three parallel process LGM’s with multi-groups (categorical moderation models) were estimated: (1) co-development between friends’ delinquency and adolescents’ delinquency for non co-offenders versus co-offenders, (2) co-development between friends’ delinquency and impulsivity for non co-offenders versus co-offenders, and (3) co-development between friends’ delinquency and school future orientation for non co-offenders versus co-offenders. Each model tested whether the slopes were correlated (i.e., “correlated change”) for the constructs of interest, as well as whether the intercepts were correlated. A Wald-test was used for the multi-group models to determine whether co-offending moderated the hypothesized co-development between adolescent delinquency and delinquency of friends, and the co-development between short-term mindsets (impulsivity and lack of school future orientation) and delinquency of friends. However, the final above-mentioned school future orientation model ran into convergence issues, and thus a similar alternative parallel LGM model was run, but without the use of a multi-group analyses. This model estimated whether the developmental trajectories (slopes) of school future orientation and delinquency of friends were correlated, as well as whether the developmental trajectories of school future orientation and co-offending were correlated. Via a Wald-test, the correlation between the slopes of school future orientation and delinquency of friends was compared to the correlation between the slopes of school future orientation and co-offending. Full maximum likelihood (FIML) estimation in Mplus, which allowed for the inclusion of variables with missing cases in the analyses (Muthén and Muthén, 1998–2017). There was a minimum of 13.7% missing data and a maximum of 31% missing data for the main variables of interest. Additionally, robust procedures (MLR) (Satorra and Bentler 1994) were employed to deal with potential non-normality. Finally, considering the large sample size, a stringent *p*-value of *p* < 0.01 was used.

## Results

### Descriptive Statistics

Tables 1 and 2 display the descriptive statistics and the correlations between the variables of interest. All variables were significantly correlated in the anticipated directions.

**Table 1:** Descriptive statistics of the variables of interest

	T1 Impuls	T2 Impuls	T3 Impuls	T1 SFO	T2 SFO	T3 SFO	T1 Co-off	T2 Del Co-off	T3 Del Co-off	T1 Del Friend	T2 Del Friend	T3 Del Friend	T1 Del	T2 Del	T3 Del
Mean	2.296	2.370	2.335	3.236	3.160	3.161	1.225	1.400	1.086	0.102	0.102	0.082	0.070	0.081	0.071
SD	0.618	0.567	0.600	0.612	0.614	0.596	0.664	0.465	0.390	0.212	0.202	0.186	0.126	0.132	0.116
Min	1.00	1.00	1.00	1.00	1.00	1.00	1.00	1.00	1.00	0.00	0.00	0.00	0.00	0.00	0.00
Max	4.00	4.00	4.00	4.00	4.00	4.00	6.00	5.00	5.50	1.00	1.00	1.00	1.00	1.00	0.86

*SFO* school future orientation, *co-off* co-offending, *Impuls* impulsivity, *Del* delinquency, *Del friend* delinquency of friends, *Min* minimum, *Max* maximum

**Table 2:** Correlations between variables of interest

	1.	2.	3.	4.	5.	6.	7.	8.	9.	10.	11.	12.	13.	14.	15.
1. T1 Impulsivity	–														
2. T2 Impulsivity	0.295**	–													
3. T3 Impulsivity	0.235**	0.390**	–												
4. T1 SFO	−0.240**	−0.129**	−0.129**	–											
5. T2 SFO	−0.153**	−0.215**	−0.153**	0.432**	–										
6. T3 SFO	−0.144**	−0.136**	−0.253**	0.364**	0.459**	–									
7. T1 Co-offending	0.143**	0.105**	0.079**	−0.217**	−0.081**	−0.029	–								
8. T2 Co-offending	0.052	0.111**	0.072*	−0.139**	−0.138**	−0.069*	0.406**	–							
9. T3 Co-offending	−0.003	0.080**	0.068*	−0.097**	−0.040	−0.092**	0.271**	0.447**	–						
10. T1 Del of friend	0.154**	0.100**	0.086**	−0.188**	−0.113**	−0.115**	0.236**	0.175**	0.091**	–					
11. T2 Del of friend	0.088**	0.096**	0.131**	−0.145**	−0.182**	−0.166**	0.172**	0.310**	0.118**	0.361**	–				
12. T3 Del of friend	0.120**	0.120**	0.126**	−0.124**	−0.132**	−0.180**	0.123**	0.150**	0.199**	0.237**	0.358**	–			
13. T1 Del	0.247**	0.142**	0.141**	−0.252**	−0.126**	−0.139**	0.410**	0.223**	0.110**	0.477**	0.288**	0.235**	–		
14. T2 Del	0.206**	0.249**	0.171**	−0.228**	−0.251**	−0.185**	0.273**	0.312**	0.180**	0.346**	0.407**	0.304**	0.568**	–	
15. T3 Del	0.181**	0.171**	0.218**	−0.180**	−0.196**	−0.260**	0.186**	0.178**	0.218**	0.232**	0.298**	0.485**	0.426**	0.593**	–

*Del* delinquency, *Del of friend* delinquency of friends, *SFO* school future orientation

*p < 0.05; **p < 0.01

### Univariate LGMs

**Fig. 2:**
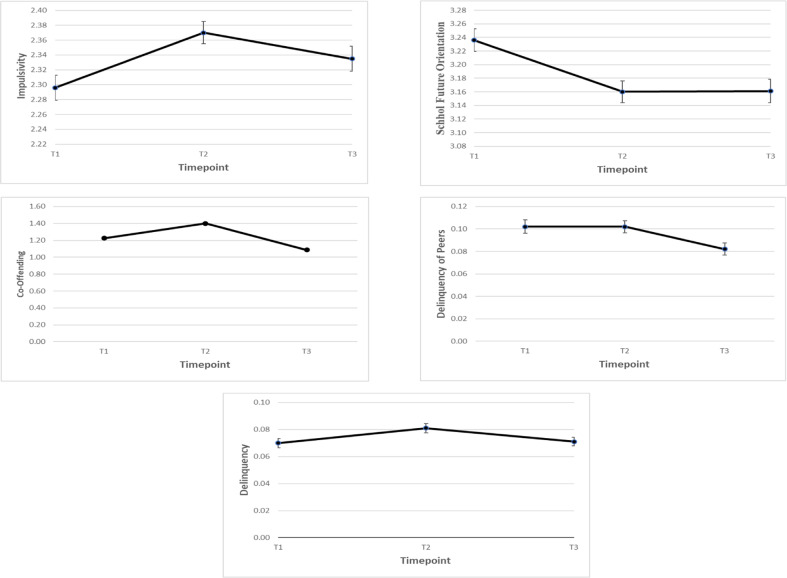
Graphs of the univariate LGM models

With the exception of the co-offending model, the non-linear models fitted better than the linear models (see Tables 3 and 4, and Fig. 2). Of note, the fit of the linear versus non-linear model of co-offending as indicated by the AIC showed negligible differences, and according to the SABIC, the linear model fitted better. Hence the linear model was retained, and it showed an excellent fit (whereas the non-linear model was just-identified).

**Table 3:** Model fit comparisons to determine the best-fitting univariate LGM models

Model	AIC	SABIC
*Impulsivity*		
Linear	7032.594	7049.590
Non-Linear	7024.768	7041.764
*School future orientation*		
Linear	6792.801	6809.796
Non-Linear	6790.316	6809.436
*Delinquency*		
Intercept only	−6463.563	−6452.941
Linear	−6524.779	−6507.783
Non-Linear	−6525.553	−6508.558
*Co-offending*		
Linear	5708.843	5725.833
Non-Linear	5708.319	5727.433
*Delinquency of friends*		
Linear	−1775.907	−1758.987
Non-Linear	−1779.058	−1762.138

The non-linear models of impulsivity, delinquency and delinquency *of* friends had a negative residual variance, which were thus constrained to zero (Muthén and Muthén, 1998–2017) to resolve estimation issues

**Table 4:** LGM Results and Model fit for the univariate best-fitting models

Measure	Intercept (variance)	(Non-)linear slope (variance)	Correlation intercept- (non-)linear slope	Chi-square (df)	CFI	TLI	RMSEA	SRMR
Impulsivity	2.297*** (0.382***)	0.059** (0.342***)	−0.814***	4.789^a^ *p* = 0.029	0.986	0.959	0.051	0.013
School Future Orientation	3.235*** (0.364)	−0.085*** (0.279)	−0.734*	0(0) *p* < 0.001	1	1	0	0
Adolescent Delinquency	0.071*** (0.016***)	0.003 (0.013***)	−0.646**	13.658^a^ *p* < 0.001	0.956	0.867	0.092	0.021
Delinquency of Friends	0.104*** (0.017***)	−0.020** (0.032***)	−0.294**	0.319^a^ *p* = 0.573	1	1.013	0	0.004
Co-offending	1.217*** (0.114**)	−0.065*** (0.012)	−0.818***	2.216^a^ *p* = 0.137	0.971	0.913	0.029	0.012

*p < 0.05; **p < 0.01; ***p < 0.001

^a^The non-linear model of future orientation was just-identified

Altogether, the results revealed that the co-offending model showed steady declines on average from T1 to T3. However, the delinquency of friends model began to level off between T1 and T2, and declined from T2 to wave T3. Thus overall there were non-linear declines on average from T1 to T3 for delinquency of friends. The delinquency of friends models showed significant variance around the intercept and slope. However, the co-offending model only showed significant variance for the intercept, but not for the slope. This indicates that whereas participants varied in their initial levels of co-offending, on average participants declined in co-offending at a similar pace.

Impulsivity showed non-linear increases from T1 to T3, as it increased from T1 to T2, and began to level off from T2 to T3. School future orientation showed the opposite pattern. That is, overall there were non-linear declines on average from T1 to T3. More specifically, it decreased from T1 to T2 and began to level off between T2 to T3. Considering the results of impulsivity and future orientation in conjunction, short-term mindsets can be said to peak at T2 (i.e., mid-adolescence) and level off thereafter. Additionally, whereas impulsivity had significant intercept and slope variance, this was not the case for school future orientation.

Finally, whereas on average adolescent delinquency remained stable throughout adolescence, there was significant intercept and slope variance. This suggests that some adolescents had upward/increasing slopes (i.e., positive slopes) in delinquency, whereas others had downward/decreasing slopes (i.e., negative slopes) in delinquency. An “intercept only model” for adolescent delinquency showed worse fit than the above-described non-linear LGM. This indicates that the slope of adolescent delinquency was needed to describe the growth in this construct. Hence the non-linear LGM of adolescent delinquency for the parallel process LGM’s was retained.

### Parallel Process LGMs

For the parallel process LGMs, the best-fitting *non-linear* LGMs for impulsivity, future orientation, delinquency of friends and adolescent delinquency were retained (see Table 3 and Fig. 2). The correlations between the growth factors are reported in Table 5a and b.

**Table 5:** Results of the (a) multi-group parallel process LGM’s of the co-development between delinquency of friends with adolescent delinquency and impulsivity (b) alternative parallel process LGM for the co-development of school future orientation, co-offending and delinquency of friends

	Delinquency of friends			
(a)	Non Co-offending Model		Co-offending Model	
	*Intercept*	*Non-linear slope*	*Intercept*	*Non-linear slope*
*Adolescent delinquency*				
Intercept	*0.458*	−0.300	0.428***	−0.364***
Non-linear slope	−0.218	0.404	−0.388**	0.652***
*Impulsivity*				
Intercept	0.246***	−0.007	*0.046*	−0.016
Non-linear slope	−0.045	−**0.051**	−0.018	**0.118**

(b)	Co-offending		Delinquency of friends	
	*Intercept*	*Linear slope*	*Intercept*	*Non-linear slope*
**School Future orientation**				
*Intercept*	−0.455***	0.424	−0.330***	0.038
*Non-Linear slope*	0.345*	−**0.438**	0.122	−**0.060**

Correlations between the slopes are depicted with bold font and correlations between the intercepts are italicized. Controlled variables included gender, educational track, ethnicity, and SES

*p < 0.05; **p < 0.01;***p < 0.001

The “adolescent delinquency and delinquency of friends” multi-group model (Chi-square (28) = 71.208, *p* < 0.001) had a moderately good fit: RMSEA = 0.053; CFI = 0.959; TLI = 0.885; SRMR = 0.028. The intercept of adolescent delinquency was positively correlated with the intercept of delinquency of friends, but only in the co-offending model. Additionally, co-development existed between adolescent delinquency and delinquency of friends. Namely, the slopes of adolescent delinquency and delinquency of friends were positively correlated in both the non co-offending model and the co-offending model. These results suggest that increases in adolescent delinquency (i.e., upward/positive slopes) were associated with increases in delinquency of friends (i.e., slower declines) in both models. Moreover, a significant moderation effect was found (Wald χ2 (1) = 22.204; *p* < 0.001), which implies that the co-development between adolescent delinquency and delinquency of friends in the co-offending model was significantly stronger than the co-development between these variables in the non co-offending model. In more substantive terms, the results showed that engaging in co-offending exacerbated the co-development between adolescent delinquency and delinquency of friends. Hence, this co-development exists at least in part because adolescents are engaging in delinquency with their friends (co-offending).

The “impulsivity and delinquency of friends” multi-group model (Chi-square (30) = 42.059, *p* = 0.071) had a very good fit: RMSEA = 0.027; CFI = 0.977; TLI = 0.940; SRMR = 0.024. For the non co-offending model, the intercepts of impulsivity and delinquency of friends were significantly and positively correlated, indicating that a higher initial level of impulsivity is related to a higher initial level of delinquency of friends. However, there were no significant correlations between these intercepts in the co-offending model. Next, the slopes of impulsivity and delinquency of friends were not significantly correlated in the non co-offending model or in the co-offending model. Thus these results do not suggest co-development between these two constructs. Finally, there was no significant moderation effect of co-offending (Wald χ2 (1) = 1.539; *p* = 0.215). This indicates that the correlations between the slopes of impulsivity and delinquency of friends did not significantly differ across the co-offending model versus the non co-offending model.

As mentioned above, the “school future orientation and friends’ delinquency” multi-group model did not converge, hence the moderation analyses were conducted in an alternative manner—without the use of a multi-group model (see Table 5b). This alternative parallel process LGM (Chi-square (29) = 73.326, *p* < 0.001) showed good fit: RMSEA = 0.035; CFI = 0.960; TLI = 0.900; SRMR = 0.022. Results showed that the intercepts of future orientation and co-offending were negatively correlated, indicating that a higher initial level of future orientation is related to a lower initial level of co-offending. Likewise, the intercepts of school future orientation and delinquency of friends were negatively correlated, indicating that a higher initial level of future orientation is related to a lower initial level of delinquency of friends. As for co-development, the slopes of school future orientation were not significantly correlated with delinquency of friends and neither with co-offending, however. No co-development was detected. Furthermore, delinquency of friends and co-offending did not interact to predict school future orientation (Wald χ2 (1) = 2.112; *p* = 0.146). In other words the correlations between the slopes of delinquency of friends and school future orientation versus the correlations between the slopes of co-offending and school future orientation did not significantly differ from each other. No moderation effect was found.

### Sensitivity Analyses

For the delinquency scale, most participants only had experience with the items that reflected minor delinquency (e.g., stealing, vandalism). The LGM models were re-run to determine whether an adjusted scale with only such minor delinquency items would alter the interpretation of the findings. This was not the case.

## Discussion

Friends’ delinquency has been consistently linked to the development of adolescents’ delinquency. However, there remain conceptual and methodological gaps in the literature on such peer delinquency in general. The current study aimed to shed light on some of these issues, while drawing upon the companions in crime hypothesis, the differential association theory, and the psychosocial maturity hypothesis. In line with these theories, the current study investigated whether delinquency shows co-development with both friends’ delinquency and short-term mindsets, and whether such co-development is moderated by co-offending. In doing so, links from ethnicity, gender, SES and educational track to the initial levels and the development of the above-mentioned variables of interest were controlled for. The hypotheses were partially supported. First, linear decreases were found in co-offending throughout adolescence, whereas there were non-linear changes in short-term mindsets which reflected a peak in such behavior during mid-adolescence. On average, no significant increases or declines in delinquency was detected, however, there was significant variance for the growth factors. As for the hypothesized co-development between these constructs, the findings revealed co-development between best friends’ delinquency and adolescents’ delinquency, and this link was stronger when adolescents had experience with co-offending. Hence a moderation effect of co-offending was present. However, there was no co-development between friends’ delinquency and short-term mindsets, and neither did co-offending moderate such hypothesized co-development. These main findings are briefly discussed below.

### Co-development of Friend’s Delinquency and Adolescent Delinquency

The development of friends’ delinquency was correlated with the development of adolescents’ delinquency. These findings are consistent with a plethora of studies on the association between peer delinquency and adolescent delinquency (see: Brechwald and Prinstein 2011). However, this appears to be among the first longitudinal studies to specifically investigate whether the co-development of the trajectories of friends’ delinquency and adolescents’ delinquency is stronger when adolescents co-offend (i.e., the companions in crime hypothesis). Nevertheless, these results concur with speculations that co-offending sets a more direct setting for learning of delinquent behaviors, which is further assumed to increase delinquency (Dynes et al. 2015). The only other similar study to also consider both delinquency of peers and co-offending, was a cross-sectional study based on 90% court-involved males and showed via moderation analyses that delinquency *of* friends is particularly associated with increased delinquency when co-offending is present (Dynes et al. 2015). The current findings extends those findings (Dynes et al. 2015) by demonstrating that this moderation effect can also be generalized to mix-gender community samples. Furthermore, the cross-sectional nature of the aforementioned study (Dynes et al. 2015) and its interaction analyses differ from the current study which examined “parallel” longitudinal co-development, which demonstrates the long-term enduring effects of these findings.

### Co-development of Friends’ Delinquency and Short-term Mindsets

The present study did not demonstrate co-development between delinquency of friends and short-term mindsets, or that co-offending moderates this co-development. Nevertheless, similar to several cross-sectional studies (Burt et al. 2006; Chapple 2005; McGloin and Shermer 2009), the current study generally did find some evidence that higher initial levels of friends’ delinquency were associated with higher initial levels of indicators of short-term mindsets. However, the non-significant longitudinal findings for the co-development between best friends’ delinquency and short-term mindsets are inconsistent with what is likely the only other longitudinal study (Goldweber et al. 2011) that investigated a similar research question. Namely, unlike the present study, it was reported that increasingly solo offenders and exclusively solo-offenders exhibited more psychosocial maturity (e.g., lower levels of short-term mindsets) than persons who at least sometimes offended in groups (i.e., “mixed-style offender”; Goldweber et al. 2011; see also: Ashton et al. 2020). Perhaps the current results differ from the abovementioned results (Goldweber et al. 2011) because of dissimilarities in sample characteristics and methodology.

Of note, the only other similar longitudinal study (Goldweber et al. 2011) was based on a sample of serious male offenders whereas the current sample included community mix-gender adolescents. Perhaps only more serious forms of co-offending is associated with short-term mindsets. Secondly, as for differences in methodology, the current analyses investigated the development of friends’ delinquency in relation to short-term mindsets with co-offending as a moderator. In contrast, the prior analyses that were used (Goldweber et al. 2011) focused on co-offending in relation to short-term mindsets, without investigating mere delinquency of peers, and neither did those analyses consider a moderating role of co-offending. Thirdly, instead of using one measurement wave of short-term mindsets and group trajectory modeling, the current analyses were based on three measurement waves of both short-term-mindsets and friends’ delinquency. Finally, the current study also controlled for multiple demographic and socioeconomic factors, which was not the case in the above-mentioned study (Goldweber et al. 2011). Thus the current methodology was more stringent in some aspects, as it took more controls into account. Such stringent controls perhaps also made it more challenging to find long-term significant effects.

Taken together, the current results suggest that co-offending exacerbates the association between friends’ delinquency and adolescents’ delinquency. Thus co-offending likely provides direct access to a setting in which individuals continue to model the delinquency they learned with their friends. The following example gives a scenario in which this can be the result. *“X has a friend Y, who vandalizes things, and X knows about it. Y then introduces X to a joint vandalizing setting (their former school) where X is exposed to vandalism, takes part in it, and learns the technique. As a result, X’s own delinquent activity increases thereafter independently of Y’s co-presence”*. However, the present results do not suggest that friends’ delinquency is also longitudinally related to short-term mindsets via co-development, despite the some base-line level correlations between these behaviors. Furthermore, similar to some other research, the current findings do not suggest that the transmission of crime-condoning tendencies of delinquent peers is dependent on co-offending (Hochstetler et al. 2002), as co-offending was not a significant moderator.

### Strengths, Limitations and Future Directions

There are also some limitations of the current study that should be mentioned. First, in (developmental) psychology, reports on delinquency of peers is the traditional method for assessing peer delinquency. However, it is important to note that adolescents might erroneously project their own delinquency onto their peers (e.g., Young et al. 2013; 2014). The “delinquency with friends” (co-offending) measure in the current study is not such a perceptual measure, as the respondents were physically present with their friends to witness the delinquency that occurred during such co-offending. Thus such a measure makes the abovementioned “projection errors” less likely or even impossible. However, the delinquency of best friends measure that was used is a perceptual measure, and it thus might be biased by the above-described “projection errors”. In any case, it is conceivable that such projection errors would be more likely among ancillary friends and less likely among best-friends relationships, which were investigated in the current study. This is because individuals in best-friend relationships are closer to each other, and therefore their perceptions of each other’s behaviors are deemed to be more reliable (for a discussion see: Rees and Pogarsky 2011). Furthermore, also of note is that what adolescents merely perceive to be true about their friends has also been shown to be decisive for predicting their behavior, independent of projection errors (Goldweber et al. 2011; Brechwald and Prinstein 2011).

Additional factors that could moderate peer influence that were not taken into account are: time spent with peers, group size of the peer networks, and peer status.

Past and more recent studies on group conformity has shown that peer influences can occur even among strangers (e.g., Asch 1951; Knoll et al. 2015). Strangers have spent no prior time together, whereas in the current study, adolescents reported on individuals they considered as their (best) friends. Hence it is reasonable to assume that the adolescent participants at least spent some time with the persons they indicated to be their (best-)friends. Additionally, a precondition for co-offending is spending time together. Thus taking prior research on peer conformity into account—since the current peer measures captured “friends” relations—it is plausible that influence processes could have occurred regardless of how much time was actually spent together between the target adolescents and their friends (for a discussion, see: Brechwald and Prinstein 2011).

As for group size, the larger the network of friends that adolescents have, the fewer time they would be able to spend with each person in their network. However, perhaps especially for deviant behavior such as delinquency, peers in broad and diffuse networks could also influence each other—independent of the size of the peer network (Brechwald and Prinstein 2011). However, of note is that larger groups have been shown to produce more violent behavior (see e.g., Lantz 2020). Although significant development links between friends’ delinquency and adolescents’ delinquency were present in the current study that did not account for group size, it would still be worthwhile for future studies—especially on co-offending—to investigate whether the size of co-offending groups further moderates peer influence effects.

A final important characteristic of the peer network that could have impacted the current results is peer status. Namely, a comprehensive review showed that peer conformity for delinquent behaviors and attitudes is stronger when the peer has a high status (Brechwald and Prinstein 2011). Also, at least one study demonstrated that older children who reported that peer-directed aggressive behavior is related to “coolness” (i.e., high peer status) showed subsequent increases in antisocial behavior during adolescence (Juvonen and Ho, 2008).

Of note, in addition to above-mentioned peer “influence” processes, just as how delinquent persons might (self-)select into delinquent peer groups (Moffitt 1993), persons with short-term mindsets might equally (self-)select into delinquent peer-groups (Chapple 2005). Furthermore, besides the abovementioned peer network characteristics, other factors such as genetics, and relatedly family relations are also important to consider in future research on peer delinquency. The robustness of the current results could further be strengthened through the use of multi-informant measures with a substantial amount of items. Namely, some of the current measures had two items which is subjected to the limitation of idiosyncratic variation (Check and Schutt 2012). Thus to overcome such limitations more items are recommended per scale (Check and Schutt 2012). Of note, although the delinquency measure that was used included many items, most adolescents only had experience with the items that reflected minor delinquency (e.g., stealing). However, the main results remained the same when the analyses were re-run with only those minor delinquency items. Finally, as for future directions, building on the significant interrelations found in the current study, future studies could further investigate mediational (e.g., via cross-lagged-panel models) and other moderating hypotheses (e.g., with peer status and/or peer group size as potential moderators).

## Conclusion

It has been put forward that co-offending could exacerbate well-established links between peer delinquency on the one hand and adolescent delinquency and short-term mindsets on the other hand. However, longitudinal studies that have investigated this hypothesis are virtually non-existent. The current study addressed this gap, and showed via its longitudinal design that co-offending is an accelerating force that links the development of best friends’ delinquency with the development of adolescents’ delinquency. Although co-offending has been known to exacerbate delinquency in real-time as groups are more violent than individuals (Lantz 2020), the current study additionally shows that co-offending has the potential to exacerbate the co-development between friends’ delinquency and adolescents’ delinquency in the long-term too. Particularly among adolescents who co-offend with friends, the development of their friends’ delinquency is more strongly related to the development of their own delinquency. Perhaps this is because co-offending with peers likely provides direct access to a crime-condoning setting, in which adolescents learn delinquency from their peers and continue to model it thereafter. Accordingly, where adolescent delinquency and its link with peer delinquency is concerned, adolescents who engage in delinquency with their peers might be worse off. This is important information for policies on co-offending and intervention programs for youth co-offenders.
